# Medical Jurisprudence, Forensic Medicine and Toxicology

**Published:** 1911-07

**Authors:** Herbert M. Hill


**Medical Jurisprudence, Forensic Medicine and Toxicology.** By **R. A. Witthaus**, A.M., M.D., Professor of Chemistry, Toxicology and Medical Jurisprudence in Cornell University ,and Tracy C. Becker, A.B., LL.D., Counsellor at Law, Professor of Criminal Law and Medical Jurisprudence in the University of Buffalo. Second edition. Vol. IV. Octavo, pp. 1271. Illustrated. New York: William Wood & Co. 1911. (Muslin, $24.00 for set; singly, $7.00 each. Half morocco, $28.00 for set; singly, $8.00 each.)

The first edition of volume IV., Medical Jurisprudence, Forensic Medicine and Toxicology by Witthaus and Becker was publiched in 1896. The volume before us, the second edition, bears the imprint of 1911. In the book before us over four hundred pages have been added to the number in the earlier work. much of this additional space being taken up with new matter or with extended detail of matters already noted before.

The plan and style of the work are for the most part exhaustive and masterly, as those who know Witthaus would expect them to be. Once in a while the author allows himself a controversial treatment of his subject which in such a work detracts from both its dignity and its value. The treatment of material is not uniformly full making it seem as though the author had lingered especially over those cases with which he himself was connected but had slighted somewhat others of equal interest to those who may use the book for reference.

We note the omission of a number of substances about which there is considerable knowledge both in the way of illustrative cases, treatment and chemical analysis, while others like hydrofluoric acid are very incomplete. Among the subjects omitted are ethyl alcohol, wood alcohol, ether, nitroglycerine, sulphonal, antipyrine, carbon bisulphide, hydrogen sulphide and tin.

The topics fully treated have a wonderful wealth of references to all aspects of the subject and show a vast amount of literary research and are fully up to date. Numerous misprints like "a solution of silver chloride" which should be a solution of silver nitrate and "potassium nitrate" which should be potassium nitrite have been corrected in this edition.

In the new matter is a list of cases referred to in the text which adds considerably to the value of the book from a lawyer's standpoint. Considerable new matter has been added in the discussion of analytical methods. Under some topics nearly every known test even the very latest is either given in full or referred to in the notes. A complete description of Thorpe's electrolytic method for arsenic with illustration of parts, a very valuable method on account of the difficulty of making arsenic free hydrogen otherwise, is given under the methods for arsenic.

As a whole, the work is the most exhaustive in the English language, is well printed and for the most part well illustrated, and this edition will prove even more valuable than its predecessor —to physician, lawyer and toxicologist.

Herbert M. Hill.

